# Supplementary material for: Small molecule regulated sgRNAs enable control of genome editing in E. coli by Cas9
Source: Nat Commun. 2020 Mar 13;11:1394. doi: 10.1038/s41467-020-15226-8 (PMC7070018; doi:10.1038/s41467-020-15226-8)
Supplement: Supplementary file 1 — Supplementary Information [file 41467_2020_15226_MOESM1_ESM.pdf]

Supplementary Discussion

Cloning and selection of the 14N library

The first limiting step of the selection was the transformation of the cloning strain with the assembled plasmid library, which yielded about 10^7 CFUs in total. The transformation of 12 µg of this subset of the plasmid library into the MGλ9 strain was expected to yield a total number of CFUs of $6x10^7$, based on control experiments with plasmids that express non-targeting gRNAs. Thus, the sub-library harvested from the E. cloni® is expected to be transformed with 99.8% completion[1] into the MGλ9 strain. However, we actually observed only $3x10^6$ c.f.u.s after transformation of MGλ9 with the subset of the plasmid library, which indicates that about 95% of the transformed plasmids probably expressed constitutively active agRNA constructs and the host cells transformed with these constructs were rapidly eliminated from the recovery culture due to cleavage of their genomic DNA.

Alternative selection step

Alternatively, the first selection step could also be performed differently, including λ-red induction. This would enable the survival of bacteria with constitutively active agRNAs but it would also lead to a *galK* knock-out. Those bacteria could then be grown in minimal M63 media that contained galactose as the only carbon source. This would starve bacteria with a *galK* knockout and eliminate constitutively active agRNAs from the selection. We propose this alternative approach can be substituted for the one described in the main text in situations where the dsDNA breaks are not lethal and do not constitute a strong selective pressure. However, for this study the use of DNA damage as selection pressure was sufficiently stringent and added less complexity to the procedure than the alternative described.

Supplementary Figures

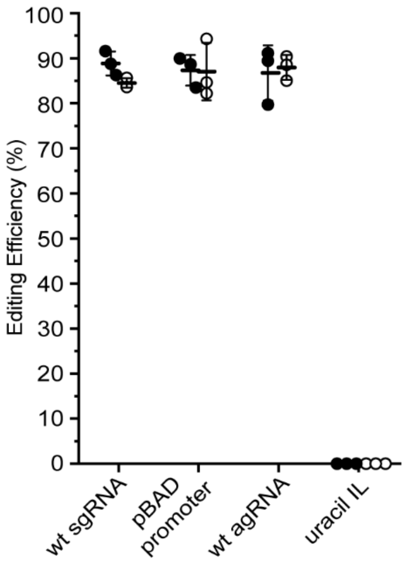

Supplementary Figure 1 Editing efficiencies at the *galK* 1 site. **wt sgRNA**: unmodified single-guide RNA. **pBAD promoter**: Cas9 is expressed from the pBAD promoter that can be induced with arabinose. Full circles: 0.2% arabinose, empty circles: no arabinose. **wt agRNA**: theophylline aptamer is inserted into the tetraloop, but 2x4 loop and upper stem are unchanged relative to wt. **uracil IL**: all 14 nucleotides of the IL and the upper stem were replaced by uracil nucleotides. Full circles: 1 mM theophylline, empty circles: no theophylline unless otherwise specified. Every circle represents one biological replicate. Error bars indicate standard deviation, horizontal bars indicate mean value. n=3 biological replicates. Source data are provided as a Source Data file.

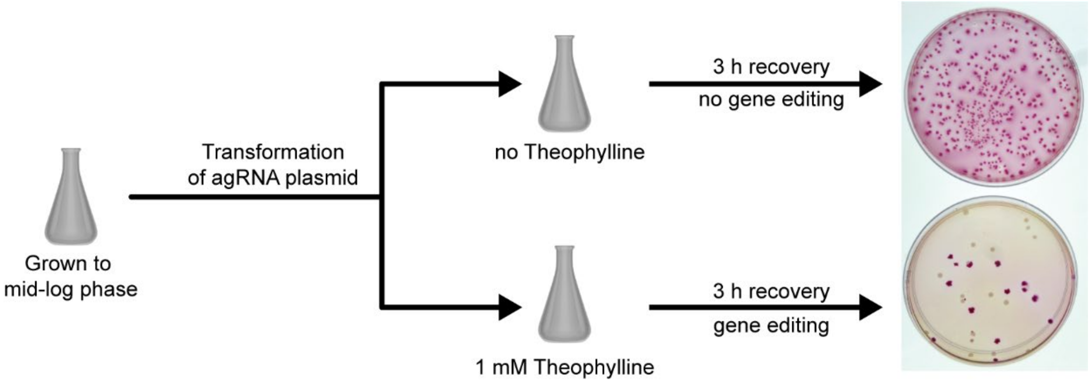

Supplementary Figure 2 Work-Flow of the screening process, based on the CREATE protocol. The bacteria are recovered for 3 hours after transformation with the aptamer ligand and then plated on MacConkey agar, containing galactose as the only source of sugar. White colonies are genomically unedited and unable to ferment galactose.

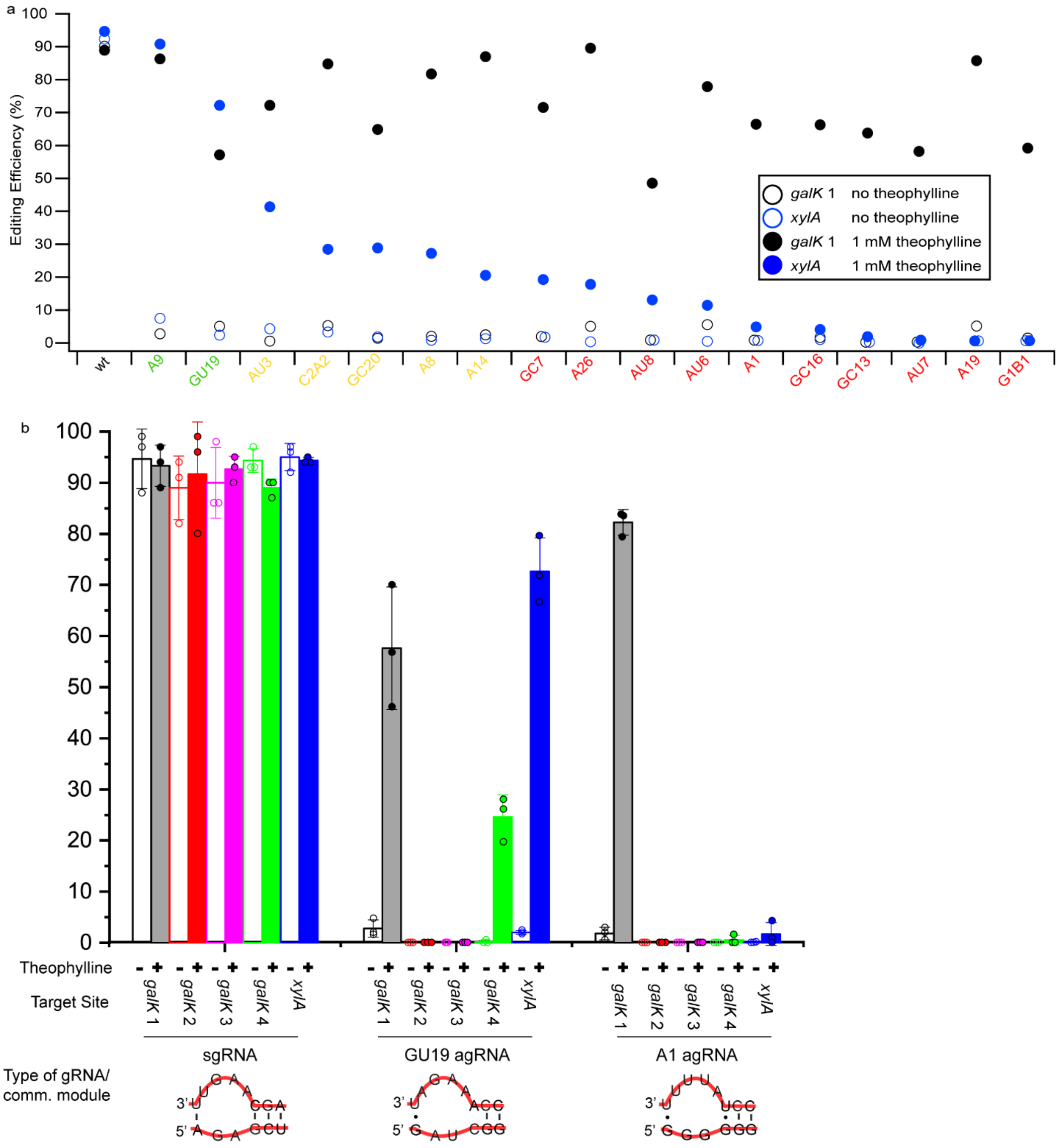

Supplementary Figure 3 Editing efficiencies of different agRNAs. **a** Gene editing at the *galK* 1 site is compared with editing at the *xylA* site for wt sgRNA and all 17 agRNA that are strongly inducible when targeting *galK* 1. **b** Additional sites at the *galK* gene were targeted with agRNA GU19 and agRNA A1 but the agRNAs could not be retargeted efficiently. Full circles: 1 mM theophylline. Empty circles: no theophylline. Bars indicate average value; error bars indicate standard deviation. n=3 biological replicates. Source data are provided as a Source Data file.

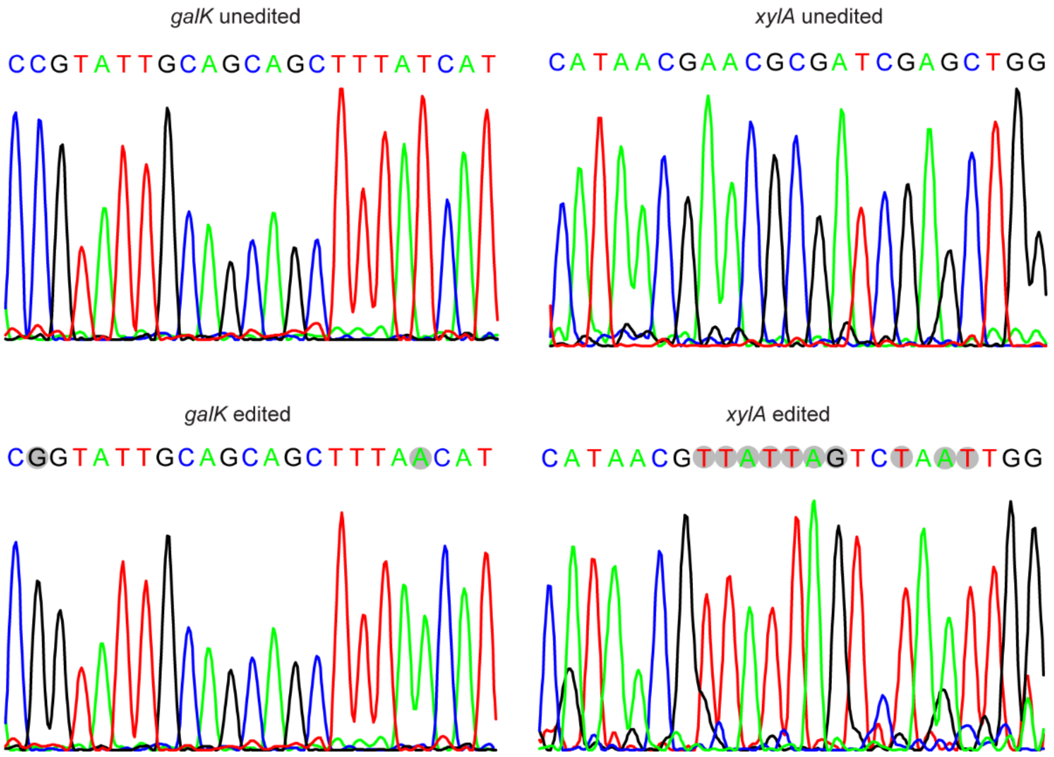

Supplementary Figure 4 Traces from Sanger sequencing after colony PCR, confirming the genomic edit in colonies with white appearance and showing no edits in colonies with red appearance. Edits correspond to the homology templates introduced for homologous recombination (Supplementary Table 2).

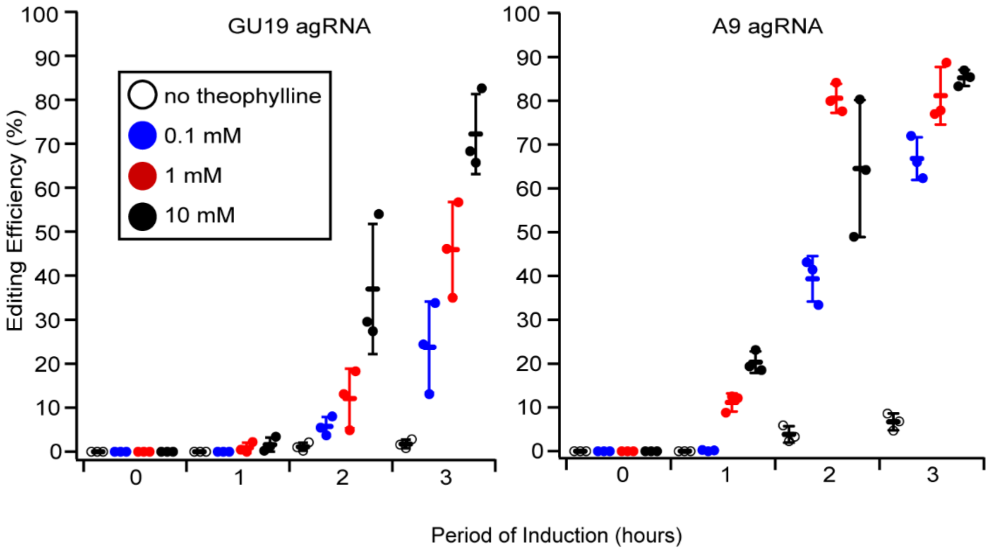

Supplementary Figure 5 This graph details the single data points summarized in Figure 2.a from the main text. The underlying data is identical. Error bars indicate +/- standard deviation from the mean. n=3 biological replicates. Source data are provided as a Source Data file.

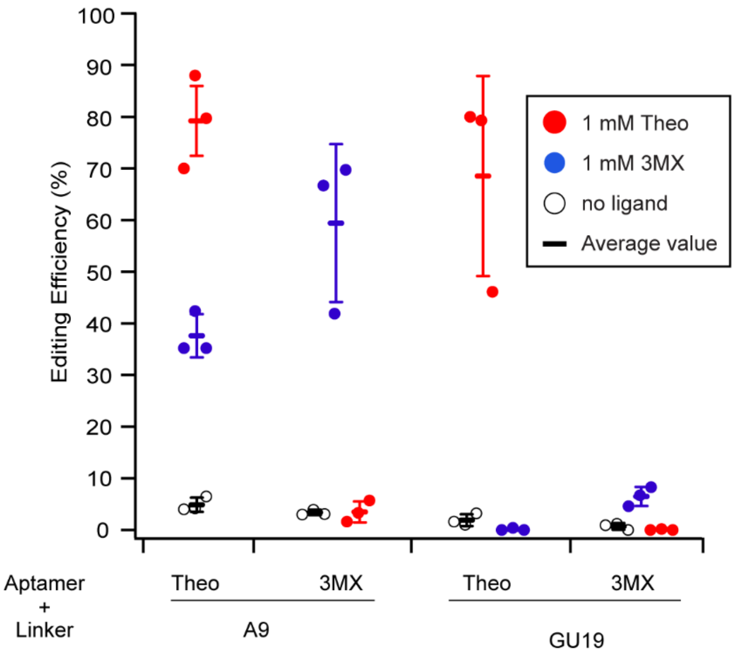

Supplementary Figure 6 This graph details the single data points summarized in Figure 2.b from the main text. The underlying data is identical. Error bars indicate +/- standard deviation from the mean. n=3 biological replicates. Source data are provided as a Source Data file.

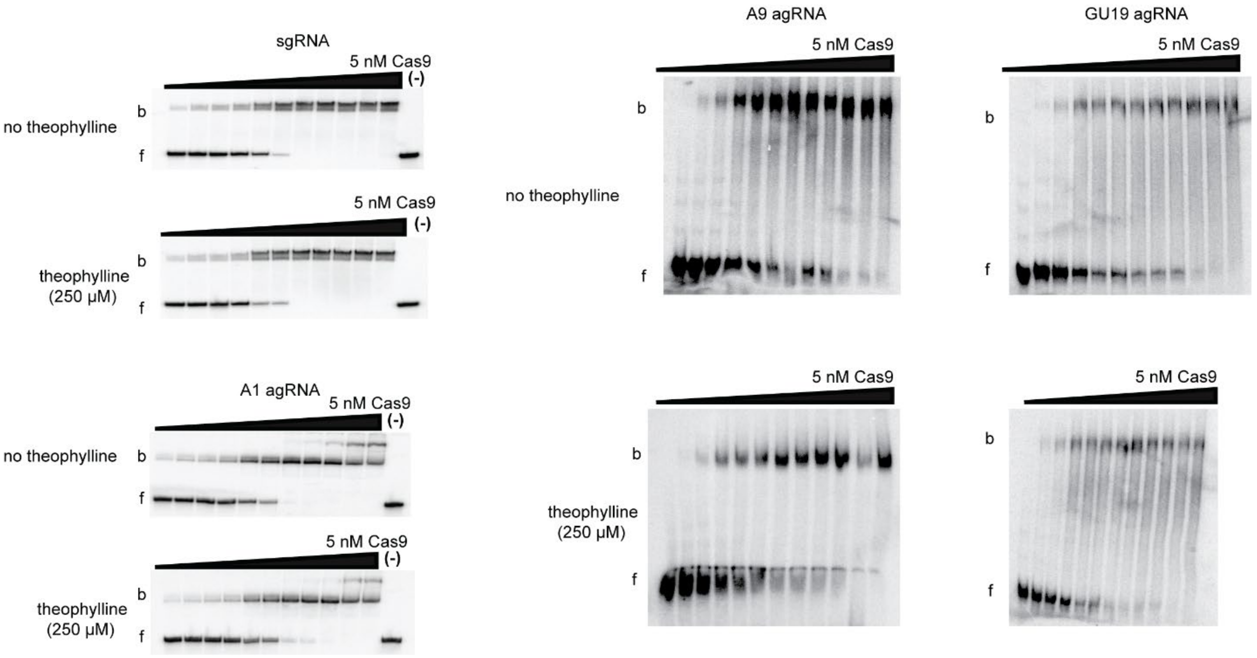

Supplementary Figure 7 Cropped images of representative gels from the electrophoretic mobility shift assay, summarized in Figure 2.c). The upper band corresponds to P^{32}-labeled agRNA bound by Cas9. The lower band corresponds to free P^{32}-labeled agRNA. Images were taken on a Typhoon FLA 9500. n=3 biological replicates. Source data are provided as a Source Data file.

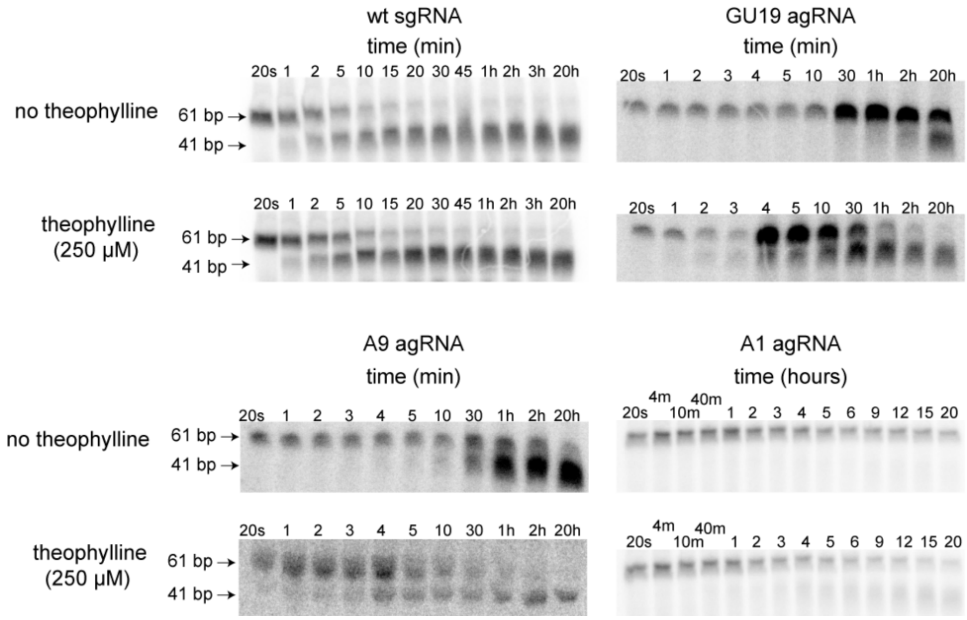

Supplementary Figure 8 Cropped images of representative gels from the *in vitro* endonuclease assay, summarized in Figure 2.d). The upper band corresponds to uncut P^{32}-labeled DNA. The lower band corresponds to nuclease products. Images were taken on a Typhoon FLA 9500. n=3 biological replicates. Source data are provided as a Source Data file.

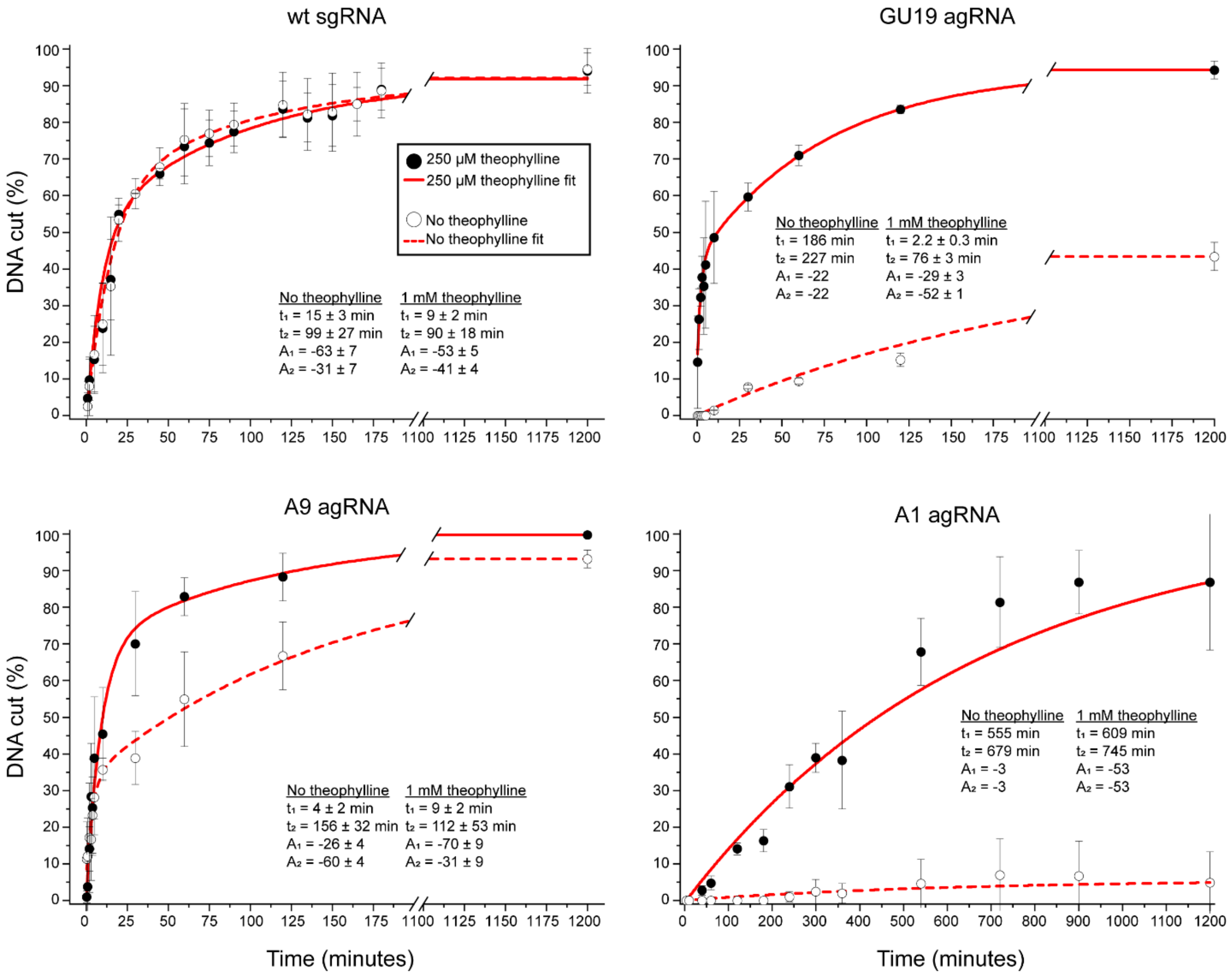

Supplementary Figure 9 Kinetics of endonuclease activity *in vitro*. 60bp ^{32}P-labeled DNA targets were cut by assembled Cas9-sgRNA RNPs and the products were resolved on a polyacrylamide gel. A two-term exponential model was fit to the data. Error bars indicate standard deviation from the mean. n=3 biological replicates. Source data are provided as a Source Data file.

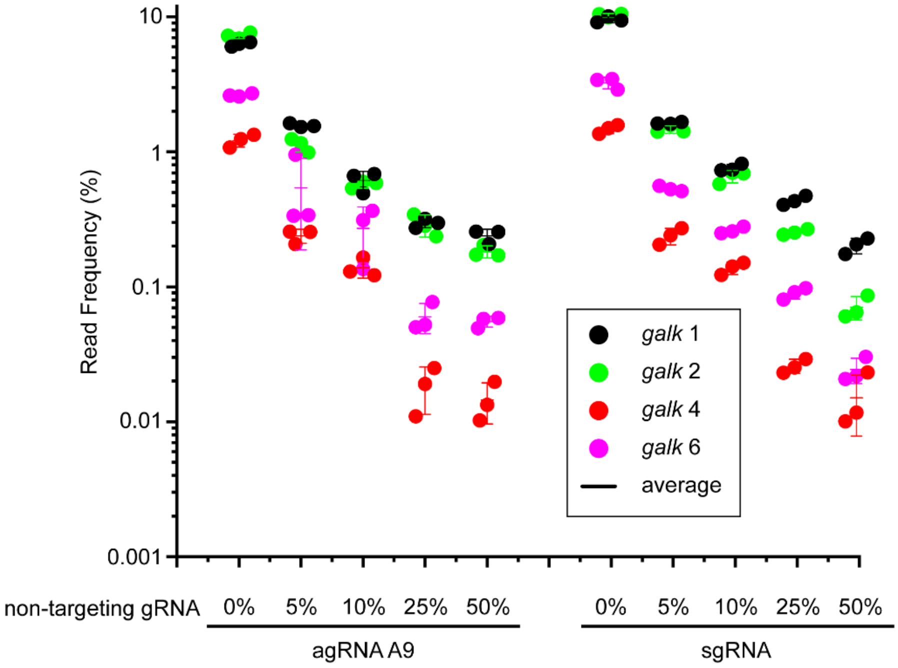

Supplementary Figure 10 Illumina sequencing results after genome editing. The frequency of targeted edits is displayed when editing with agRNAs or sgRNAs. The plasmids transformed were an equimolar mix of four plasmids that express agRNAs or sgRNAs that target one site on the *galK* gene each. Additionally, plasmids expressing non-targeting sgRNAs were added to varying molar percentages. Error bars indicate +/- standard deviation from the mean. n=3 biological replicates. Source data are provided as a Source Data file.

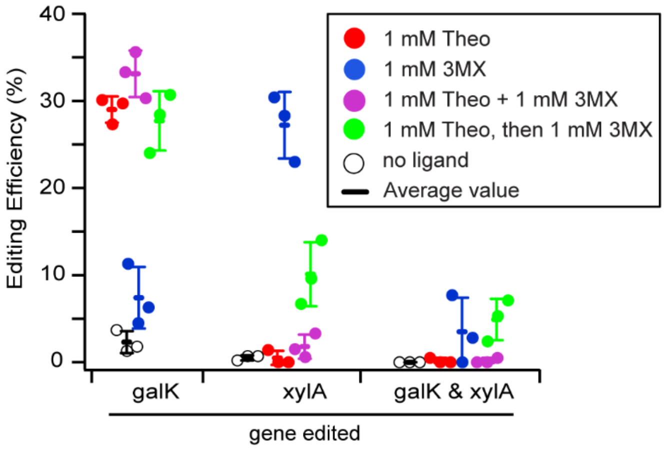

Supplementary Figure 11 This graph details the single data points summarized in Figure 3.e from the main text. The underlying data is identical. Error bars indicate +/- standard deviation from the mean. n=3 biological replicates. Source data are provided as a Source Data file.

Supplementary Tables

Supplementary Table 1

All sequence cassettes for the 2x4 IL and upper stem that were sequenced and tested are listed below with their respective editing efficiency at the *galK* 1 site when induced for 3 hours with 1mM theophylline. *±SD represents the standard deviation from the mean. **The mean value was calculated from three biological replicates

		**Editing Efficiency**			
**Name**	**Sequence**	**no Theo**	**±SD***	**1 mM Theo**	**±SD***
sgRNA	AGAGCTAGCAAGTT	85%**	1%	89%**	3%
**Casettes from initial Library**					
G1B1	GTATCGCTTAAGCC	1%		59%	
RG53	AGTGAGCTAAAAAT	0%		22%	
A38	GAGAGGCCCCCGGC	5%		85%	
A9	TGAAGGCCGCAACA	9%**	7%	81%**	7%
A39	AGAAGGCCCATCAT	0%		7%	
A34	TAGTTTAAACCGTT	12%		57%	
A1	GGGGGGCCTATTTT	0%		66%	
A10	GATGGGCCTCCACC	0%		14%	
A14	GGAGGTACGGTGCC	2%		87%	
A19	GAGAGGCCCCCGGC	20%		90%	
A8	CGGGGGACAATAGG	2%		82%	
A26	GGGGGGCCACGCGC	5%		89%	
C2A2	TAGTGGCTACCATG	5%		84%	
**Casettes from optimized libraries**					
AU1	AGGGGGCCTATAAT	0%		2%	
AU2	ACCAGGCCAAGTAT	0%		4%	
AU3	ACAAGGCCCATAAT	1%		72%	
AU4	AACAGGCCTTAATT	0%		0%	
AU5	ACTCGGCCTGAACT	0%		43%	
AU6	AAGGGGCCTATAAT	5%		77%	
AU7	AGTAGGCCTTTCAT	0%		58%	
AU8	ACGGGGCCTAATAT	0.3%		48%	
AU9	AAACGGCCCACTGT	1%		22%	
AU10	ATAGGGCCATCCAT	0%		13%	
AU11	AATAGGCCACTTAT	0%		25%	
AU12	AGAGGGCCGGGCGT	0%		26%	

AU14	AGTGGGCCAGCCTT	0%		17%	
AU15	ACCCGGCCATTCAT	0%		5%	
AU16	AACCGGCCCCGAGT	0%		6%	
AU17	AAAGGGCCAGGCAT	0%		4%	
AU18	AATAGGCCCAGACT	0%		7%	
AU19	AATAGGCCCGCAGT	0%		10%	
AU20	AAATGGCCGGCAAT	0%		0%	
GU1	GACGGGCCTAATAT	0%		0%	
GU2	GCGAGGCCTACTAT	0%		0%	
GU3	GCAGGGCCTCATTT	0%		0%	
GU4	GATTGGCCATACAT	0%		0%	
GU5	GGTTGGCCTAATAT	0%		2%	
GU6	GGACGGCCAAGCAT	0%		1%	
GU8	GGCAGGCCTCTTCT	0%		1%	
GU9	GTTCGGCCCCGACT	0%		0%	
GU10	GTACGGCCCATAAT	0%		0%	
GU11	GTATGGCCTCGGAT	0%		0%	
GU12	GCCGGGCCTTTTTT	0%		7%	
GU13	GGCCGGCCAAGCAT	0%		6%	
GU14	GACTGGCCTATAAT	0%		2%	
GU15	GGACGGCCTACAAT	0%		11%	
GU16	GGGAGGCCAGCGAT	1%		0%	
GU17	GTAAGGCCGCCGAT	0%		0%	
GU18	GATTGGCCTACGGT	0%		1%	
GU19	GATCGGCCATAGAT	3%**	3%	64%**	4%
GC1	GCTGGGCCCTTCCC	0%		0%	
GC2	GTCAGGCCTATACC	0%		0%	
GC3	GTCCGGCCCCACAC	0%		12%	
GC4	GGTCGGCCAGTAGC	0%		1%	
GC5	GTTGGGCCGCGGAC	0%		0%	
GC6	GATTGGCCAGCAAC	1%		40%	
GC7	GGGGGGCCGAATAC	1%		71%	
GC8	GGACGGCCCTGTGC	1%		0%	
GC9	GCAGGGCCTCTAAC	0%		0%	
GC10	GAGCGGCCACAACC	0%		0%	
GC11	GCGTGGCCCTTCCC	0%		3%	
GC12	GATAGGCCAGTTAC	1%		36%	
GC13	GGAAGGCCTTATAC	0%		64%	
GC15	GCATGGCCTACTCC	1%		21%	

GC16	GATAGGCCAACACC	1%		65%	
GC17	GACCGGCCCCCCGC	0%		2%	
GC18	GATTGGCCGCAACC	0%		48%	
GC20	GCAAGGCCAACACC	0%		64%	

Supplementary Table 2

DNA and RNA Sequences and oligonucleotides used in this study.

Name	Sequence
HA_rev	agatctttagaattccagaaatcatc
CREATEBB no gRNA2	GAAGCTTGGGCCCGAACAAAAAC
tracrRNA_rev	CAAGTTGATAACGGACTAGCCTTATTTT
galK spacer BB rev	CTATTGCAGCAGCTTTATCATAC
A9 bb_fwd	GAAGGATACCAGCTTCGAAAG
A9 bb_rev	AGATCTTTAGAATTccagaaatcatcc
theo_insert_B 1	GGTATAATACTAGTATGATAAAGCTGCTGCAATAGTTTTNNNNNNATACCAGCT TCGAAAGAAGCCCTTGGCAG
theo_insert_B 2	TTTTTCAAGTTGATAACGGACTAGCCTTATTTTNNNNNNNNCTGCCAAGGGCTT CTTTCGAAGCTG
theo-gRNA_bbF	AAAACTATTGCAGCAGCTTTATCATACTAGTATTATACC
theo-gRNA_bbR	AAAATAAGGCTAGTCCGTTATCAACTTGAAAAAG
agRNA from Liu et al.[2]	ATGATAAAGCTGCTGCAATAGTTTTAGAGCTAGAAATAGCAAGTTAAAATAAGG CTAGTCCGTTATCAACTTGAAAAAGTGGCACCGAGTCGGTGCCAGCAGCTTTAT CATATACCACGCGAAAGCGCCTTGGCAGATGATAAAGTTTTTTT
CREATESeq _fwd	CTAAGGATGATTTCTGGAATTC
CREATESeq _rev	CAGAACGCAGAAGCG
Nextera Adapter R1	TCGTCGGCAGCGTCAGATGTGTATAAGAGACAGAGTTTTCCCTCGATGCGCCC
Nextera Adapter R2	GTCTCGTGGGCTCGGAGATGTGTATAAGAGACAGTCGCTGCCAACCAGGGTACG
In vitro target	GATTTGTGCGCCGTCCAGCGGCAGATGATAAAGCTGCTGCAATACGGTTCCGAC CGCGACT
Theophylline aptamer	AUACCAGCUUCGAAAGAAGCCCUUGGCAG
3-methylxanthine aptamer	AUACCAGCUUCGAAAGAAGCC**A**UUGGCAG

guides of the gRNAs	Sequence
galK 1	AUGAUAAAGCUGCUGCAAUA
galK 2	GAUCAGCGGCAAUGUGCCGC
galK 3	GUUCACCAAUCAAAUUCACG
galK 4	GACCGCGACUUCCAGUGAAG
galK 6	GCAGCTTTATCATCTGCCGC
xylA	CAUAACGAACGCGAUCGAGC
eGFP (non-targeting)	GACCAGGAUGGGCACCACCC
mCherry	CCAAGCTGAAGGTGACCAA
lacZ 1	TCGCGGTGATGGTGCTGCGC
lacZ 2	CGGATAATGCGAACAGCGCA
lacZ 3	ATTTCGCTGGTGGTCAGATG
ispC	TTCCAGACCTTTGTTCATCA
**Homology Templates**	**Sequence**
galK 1	CCGCAGGGTGCCGGGTTAAGTTCTTCCGCTTCACTGGAAGTCGCGGTCGGAACGGTATTGCAGCAGCTTTAACATCTGCCGCTGGACGGCGCACAAATCGCGCTTAACGGTCAGGAAGCA
galK 2	GAGCACCGCCTTCGTCTGTCGAGATAGGACATCTGCAACTGCGTAACAACAGCTTCGGCGGCGTGGACATGGTGATCAGCGGCTAATAACCACAAGGTGCCGGGTTAAGTTCTTCCGCTTCACTGGAAGTCGCGGTCGGAACCGTA
galK 3	GAGCACCGCCTTCGTCAGATCTGTAGTCCTCTGTTTGCCAACGCATTTGGCTACCCTGCCACTCACACCATTCAGGCGCCTGGCCGTGTTTAATAAATTGGTGAACACACCGACTACAACGACGGTTTCGTTCTGCCCTGCGCGAT
galK 4	AAGAGCACCGCCTTCGTCGCTAAGTCTGACTCGGCGGCGTGGACATGGTGATCAGCGGCAATGTGCCGCAGGGTGCCGGGTTAAGTTCTTAATAAAGCCTGGAAGTCGCGGTCGGAACCGTATTGCAGCAGCTTTATCATCTGCCGC
galK 6	TACGAGAGGATCCCGCTTCACTGGAAGTCGCGGTCGGAACCGTATTGCAGCAGCTTTATCATTTACCATTATAATAAGCACAAATCGCGCTTAACGGTCAGGAAGCAGAAAACCAGTTTGTAGGCTGTAA
xylA	CATCACCCGCGGCATTACCTGATTATGGAGTTCAATATGCAAGCCTATTTTGACCAATTAGACTAATAACGTTATGAAGGCTCAAAATCCTCAAACCCGTTAGCATTCCGTCACTACA

lacZ 1	CCTACTGTACGTTCGGTTACGGCCAGGACAGTCGTTTGCCGTCTGAATTTGACC<br>TGAGCGCATTTTTACGCGCCGGAGAAAACCGCCTCGCGGTGATGGTGTAATAAT<br>GGAGTGACGGCAGTTATCTGGAAGATCAGGATATGTGGCGGATGAGCGGCATTT<br>TCCGTGACGTCTCGTTGCTGCATAAACCGACTACA
lacZ 2	TAACATGCGTACTGGGTTTCAATATTGGCTTCATCCACCACATACAGGCCGTAG<br>CGGTCGCACAGCGTGTACCACAGCGGATGGTTCGGATAATGTTATTAGCGCACC<br>GCGTTAAAGTTGTTCTGCTTCATCAGCAGGATATCCTGCACCATCGTCTGCTCA<br>TCCATGACCTGACCATGCAGAGGATGATGCTCGTG
lacZ 3	CGATCAAGCTACACATCTGTGAAAGAAAGCCTGACTGGCGGTTAAATTGCCAAC<br>GCTTATTACCCAGCTCGATGCAAAAATCCATTTCGCTGGTGGTTTATTATGGGA<br>TGGCGTGGGACGCGGCGGGGAGCGTCACACTGAGGTTTTCCGCCAGACGCCACT<br>GCTGCCAGGCGCTGATGTGCCCGGCTTCTGACCAT
ispC	CCGCGAGGTTTGCAGGGTCCTGCCGTCATCCGAACTGGTCGATGGGGCGTAAAA<br>TTTCTGTCGATACCGCTACGATGATGAACAAAGGTCTGGAATACATTGAAGCGC<br>GTTGGCTGT

Supplementary Table 3 The over-representation of plasmids expressing non-targeting gRNAs was confirmed by colony PCR and Sanger sequencing. 20 ng CREATE plasmids expressing a non-targeting gRNA were co-transformed with 180 ng of CREATE plasmid expressing a targeting gRNA construct and recovered with 1 mM theophylline. For each of those experiments, 16 colonies that showed up as unedited on the MacConkey agar were picked. For the experiments with A9 and GU19, the different sizes of the non-targeting wt sgRNA and the targeting agRNAs were used for differentiation via agarose gel electrophoresis after colony PCR. For the experiment with targeting and non-targeting wt sgRNA, Sanger sequencing was used to determine the identity of the transformed plasmid.

gRNA construct co-transformed with non-targeting gRNA	# of unedited colonies carrying non-targeting gRNA plasmid
A9 agRNA	9/16
GU19 agRNA	6/16
wt sgRNA	16/16

References

1. Bosley, A. D. & Ostermeier, M. Mathematical expressions useful in the construction, description and evaluation of protein libraries. *Biomol. Eng.* **22**, 57–61 (2005).
2. Liu, Y. *et al.* Directing cellular information flow via CRISPR signal conductors. *Nat. Methods* **13**, 938–944 (2016).
