# Supplementary material for: Small molecule regulated sgRNAs enable control of genome editing in E. coli by Cas9
Source: Nat Commun. 2020 Mar 13;11:1394. doi: 10.1038/s41467-020-15226-8 (PMC7070018; doi:10.1038/s41467-020-15226-8)
Supplement: Supplementary file 2 — Reporting Summary [file 41467_2020_15226_MOESM2_ESM.pdf]

nature research

Corresponding author(s): Robert Batey

Last updated by author(s): Feb 13, 2020

Reporting Summary

Nature Research wishes to improve the reproducibility of the work that we publish. This form provides structure for consistency and transparency in reporting. For further information on Nature Research policies, see Authors & Referees and the Editorial Policy Checklist .

Statistics

For all statistical analyses, confirm that the following items are present in the figure legend, table legend, main text, or Methods section.

n/a	Confirmed	
☐	☒	The exact sample size (n) for each experimental group/condition, given as a discrete number and unit of measurement
☐	☒	A statement on whether measurements were taken from distinct samples or whether the same sample was measured repeatedly
☒	☐	The statistical test(s) used AND whether they are one- or two-sided<br>*Only common tests should be described solely by name; describe more complex techniques in the Methods section.*
☒	☐	A description of all covariates tested
☒	☐	A description of any assumptions or corrections, such as tests of normality and adjustment for multiple comparisons
☐	☒	A full description of the statistical parameters including central tendency (e.g. means) or other basic estimates (e.g. regression coefficient) AND variation (e.g. standard deviation) or associated estimates of uncertainty (e.g. confidence intervals)
☒	☐	For null hypothesis testing, the test statistic (e.g. F, t, r) with confidence intervals, effect sizes, degrees of freedom and P value noted<br>*Give P values as exact values whenever suitable.*
☒	☐	For Bayesian analysis, information on the choice of priors and Markov chain Monte Carlo settings
☒	☐	For hierarchical and complex designs, identification of the appropriate level for tests and full reporting of outcomes
☒	☐	Estimates of effect sizes (e.g. Cohen's d, Pearson's r), indicating how they were calculated

Our web collection on statistics for biologists contains articles on many of the points above.

Software and code

Policy information about availability of computer code

Data collection	No software was used for data collection.
Data analysis	ImageQuant TL and ImageJ 1.52a were used to quantify images of the radiographs. Igor Pro8 and Origin 2018b were used for fitting models to the EMSA and nuclease assay data. ProtParam was used to calculate the extinction coefficient of Cas9.

For manuscripts utilizing custom algorithms or software that are central to the research but not yet described in published literature, software must be made available to editors/reviewers. We strongly encourage code deposition in a community repository (e.g. GitHub). See the Nature Research guidelines for submitting code & software for further information.

Data

Policy information about availability of data

All manuscripts must include a data availability statement. This statement should provide the following information, where applicable:

- Accession codes, unique identifiers, or web links for publicly available datasets
- A list of figures that have associated raw data
- A description of any restrictions on data availability

The authors declare that the main data supporting the findings of this study are available within the article and its supplementary information files. The source data for figures 1d, e, 2a-d, 3b, c, e, f and supplementary figures 1, 3a, b, 5-8, 10, 11 are provided as a Source Data File.

Field-specific reporting

Please select the one below that is the best fit for your research. If you are not sure, read the appropriate sections before making your selection.

Life sciences study design

All studies must disclose on these points even when the disclosure is negative.

Sample size	n=3-5. This sample size was chosen because preliminary experiments showed that statistical significance is typically reached at these sample sizes. For counting colonies on agar plates, we aimed for 300-500 colonies per plate as this is the highest number possible while maintaining distinct colonies.
Data exclusions	No data was excluded
Replication	n=3-5 for most data shown. Experimental replicates were biological replicates, often collected on different days and sometimes by different authors. Replication of the findings was successful. n=1 only for the preliminary screening process in Supplementary Figure 3a.
Randomization	We did not work with various biological strains, so randomization of subjects/samples was not applicable.
Blinding	We did not work with various biological strains, so blinding was not applicable.

Reporting for specific materials, systems and methods

We require information from authors about some types of materials, experimental systems and methods used in many studies. Here, indicate whether each material, system or method listed is relevant to your study. If you are not sure if a list item applies to your research, read the appropriate section before selecting a response.

Materials & experimental systems

n/a	Involved in the study
☒	☐ Antibodies
☒	☐ Eukaryotic cell lines
☒	☐ Palaeontology
☒	☐ Animals and other organisms
☒	☐ Human research participants
☒	☐ Clinical data

Methods

n/a	Involved in the study
☒	☐ ChIP-seq
☒	☐ Flow cytometry
☒	☐ MRI-based neuroimaging
